# Thymic Epithelial Tumors as a Model of Networking: Development of a Synergistic Strategy for Clinical and Translational Research Purposes

**DOI:** 10.3389/fonc.2020.00922

**Published:** 2020-07-14

**Authors:** Enrico Melis, Enzo Gallo, Simona di Martino, Filippo Tommaso Gallina, Valentina Laquintana, Beatrice Casini, Paolo Visca, Federica Ganci, Gabriele Alessandrini, Mauro Caterino, Fabiana Letizia Cecere, Chiara Mandoj, Arianna Papadantonakis, Nicoletta De Bello, Rossano Lattanzio, Giovannella Palmieri, Marina Chiara Garassino, Nicolas Girard, Laura Conti, Giovanni Blandino, Francesco Fazi, Francesco Facciolo, Edoardo Pescarmona, Gennaro Ciliberto, Mirella Marino

**Affiliations:** ^1^Thoracic Surgery Unit, IRCCS Regina Elena National Cancer Institute, Rome, Italy; ^2^Department of Pathology, IRCCS Regina Elena National Cancer Institute, Rome, Italy; ^3^Oncogenomic and Epigenetic Unit, IRCCS Regina Elena National Cancer Institute, Rome, Italy; ^4^Radiology Unit, IRCCS Regina Elena National Cancer Institute, Rome, Italy; ^5^Medical Oncology 1, IRCCS Regina Elena National Cancer Institute, Rome, Italy; ^6^Clinical Pathology, IRCCS Regina Elena National Cancer Institute, Rome, Italy; ^7^University “G. d'Annunzio,” Department of Medical, Oral and Biotechnological Sciences, Center for Advanced Studies and Technology (CAST), Chieti, Italy; ^8^Scientific Direction, Department of Clinical Medicine and Surgery, Rare Tumors Reference Center, University Federico II, Naples, Italy; ^9^Thoracic Oncology Unit, Division of Medical Oncology, Foundation IRCCS–Italian National Cancer Institute, Milan, Italy; ^10^Institut du Thorax Curie-Montsouris, Institut Curie, Paris, France; ^11^Department of Anatomical, Histological, Forensic & Orthopedic Sciences, Section of Histology & Medical Embryology, Sapienza University of Rome, Laboratory Affiliated to Instituto Pasteur Italia-Fondazione Cenci Bolognetti, Rome, Italy; ^12^Scientific Direction, IRCCS Regina Elena National Cancer Institute, Rome, Italy

**Keywords:** thymic epithelial tumors, thymoma, thymic carcinoma, biobank, microRNA, TCGA, ITMIG, EURACAN

## Abstract

Among the group of thymic epithelial tumors (TET), thymomas often show either uncertain or explicit malignant biological behavior, local invasiveness, and intrathoracic relapse and are often difficult to manage. From the initial stages, thymic carcinomas tend to show aggressive behavior and extrathoracic spread. Moreover, the interplay of epithelial cells and thymocytes in thymomas causes complex immune derangement and related systemic autoimmune diseases. Due to their rare occurrence and to the limited funding opportunities available for rare tumors, it is challenging to make advances in clinical and translational research in TET. The authors of this paper are all members of a multidisciplinary clinical and research thoracic tumor team. Strong input was given to the team by long-standing expertise in TET in the Pathology Department. In addition, thanks to the collaboration between research units at our Institute as well as to national collaborations, over the last 10 years we were able to perform several tissue-based research studies. The most recent studies focused on microRNA and on functional studies on the thymic carcinoma cell line 1889c. The recent implementation of our biobank now provides us with a new tool for networking collaborative research activities. Moreover, the participation in a worldwide community such as ITMIG (International Thymic Malignancy Interest Group) has allowed us to significantly contribute toward fundamental projects/research both in tissue-based studies (The Cancer Genome Atlas) and in clinical studies (TNM staging of TET). Our achievements derive from constant commitment and long-standing experience in diagnosis and research in TET. New perspectives opened up due to the establishment of national [the Italian Collaborative Group for ThYmic MalignanciEs (TYME)] and European reference networks such as EURACAN, for an empowered joint clinical action in adult solid rare tumors. The challenge we face still lies in the advancement of clinical and basic science in thymic epithelial malignancies.

## Introduction

Thymic epithelial tumors (TET) are a rare group of tumors, comprising thymoma (THY) and thymic carcinoma (TC), that have an incidence rate of 0.13/100,000 per population in the United States according to the National Cancer Institute's (NCI) Surveillance, Epidemiology, and End Results (SEER) program/SEER database (DB) (1). Population-based data were provided by the European cancer registries (CRs) participating in the RARECARE project: compared to that in the United States, TET showed a slightly higher incidence rate of 0.17/100,000 per population, and “malignant” thymomas accounted for 0.14/100,000. TC, a much rarer disease than THY, occurs with an incidence rate of 0.2–0.5 per million individuals (2). Data on the epidemiology of two families of rare thoracic neoplasias (epithelial tumors of thymus and mesothelioma of pleura and pericardium) for 27 European countries have been recently reported in more detail by the RARECARENet working group (www.rarecarenet.eu) Malignant TET showed (in the period 2000–2007) a 5-year survival of 64%, on average (3). Recent advances in tumor biology and pathology reveal that TET constitute a unique group of neoplasias deriving from the epithelial cell network of the thymus (TEC). The extraordinary properties and characteristics of this primary lymphatic organ have been firmly established in the last 60 years, after the discovery by Miller (4) and Good (5) of the unique thymic immunological functions. Due to its central role in the homeostasis of the immune system, it is not surprising that the tumors deriving from TEC are associated with derangement of the immune system (6). In 2015, the World Health Organization (WHO) changed the International Classification for Disease of Oncology (ICD-O) code associated with thymoma from the suffix /1 applied to the third edition classification (7) to the suffix /3 for the fourth edition (8). This change reflects our increased knowledge in the biology of TET and contributes to forming the statement that “all thymomas can behave in a clinically aggressive fashion” irrespective of tumor stage and should be considered malignant (9). In recent years, significant interest in TET has been shown all around the world, and much progress has been made in the last few years due to the activity of the International Thymic Malignancy Interest Group (ITMIG) scientific society (10, 11) (www.itmig.org) and to the International Association for the Study of Lung Cancer (IASLC). Due to the joint effort of ITMIG and of IASLC and to the contribution of several important DBs (12), TET for the first time were included in the TNM staging system (13). The new staging system relies on retrospective data from more than 10,000 TET cases observed all around the world (14). Specific interest raised toward these unique tumors was also due to the US NCI's inclusion of TET in The Cancer Genome Atlas (TCGA) project (15), only one of the few families of rare tumors considered. Moreover, due to the inclusion of TET in the rare cancers included in the G8 group (rare thoracic tumors) of EURACAN, the network of rare adult solid cancers in the European reference networks (ERNs) (http://euracan.ern-net.eu), significant progress in their management has to be expected over the next few years (16). Recently, we also joined the Italian Collaborative Group for ThYmic MalignanciEs (TYME) as a reference center for the diagnosis and treatment of TET (17).

We wish to point out that the driving force behind bringing new opportunities in rare tumor research and international collaborations to our local setting was the renewed commitment and long-standing expertise of the Pathology Department. Pathology now plays a major role in bridging the gap between tumor research and clinical management in every field of tumor research. This also applies to our Institute in relation to the TET family of rare tumors. We describe here our own developing workup within the clinical and scientific contexts of TET, focusing mainly on the surgical approach, on the pathological workup, and on the ongoing research activities in different fields. Recently, a renewed opportunity was offered by progressing from a “sample collection”-based biobank to an institutionally certified ISO9001:2015 biobank. We discuss here specificities, critical issues, and challenges, focusing on our surgical, pathological, and biobank activities, as these are the main players of translational research. We also briefly mention the research projects accomplished to date and discuss how we will implement and improve our model/strategy for making progress in the future.

## Materials and Equipment

The surgical procedures cited for both open-access and mini-invasive approaches for TET were performed with the standard surgical instruments of a thoracic surgery operating room. Robotic thymectomy was performed by the da Vinci® surgical system (Intuitive Surgical Inc.).

### Laboratory Methods and Equipment at the Pathology Department

Fixation of tumor specimens in 10% buffered formalin and routine laboratory techniques and equipment of a pathology laboratory were adopted to fix and to process tumor samples. Hematoxylin–eosin (H&E) was the standard routine used for staining. The Aperio system AT2 (Aperio Leica Biosystems) (CE IVD) whole-slide scanner (400-slide capacity) was used to scan slides for digital pathology.

We mention here only the main equipment available at the Pathology Department; other platforms/equipment found in the collaborating laboratories are described in detail elsewhere (18, 19): Immunohistochemistry (IHC) at our Pathology Department is performed on BOND-III, the fully automated IHC platform (LEICA BIOSYSTEMS). Our molecular biological/genetic equipment includes (1) the Ion Gene Studio™ S5 series for next-generation sequencing (NGS, Thermo Fisher); (2) the Applied Biosystems 3130 Genetic Analyzer (Thermo Fisher). The Platforms for MicroRNA study (Agilent 2,100 Bioanalyzer and “Affymetrix® Human Gene 2.0 ST Arrays 2.0,” both from Affymetrix, Santa Clara, California) are of routine research use at our Oncogenomic and Epigenetic Research Unit.

Biobanking instruments include cryogenic systems, labeling machines, and barcode readers. Systems for cryopreservation include electric freezers (−80°C); liquid nitrogen storage systems; a dedicated biobanking software, EasyTrack2D® and instruments used for quality control of biological samples in measuring various cellular components (DNA, RNA, and protein) (Bioanalyzer, Agilent Biotechnologies). All these sets of equipment are available within the dedicated spaces with controlled access. Our biobank is ISO certified (ISO9001:2015) (20).

## Methods

### Care Pathway of TET

Between 2000 and 2019, 196 patients were recorded in our DB at the IRCCS Regina Elena National Cancer Institute (IRE), including demographic data, histologic type updated to 2015 WHO classification (8), surgical procedures, and the main outcome indicators. Cases evaluated for pathological diagnosis as a second opinion were recorded together with internal cases.

### Diagnostic/Preoperative Workup

At our Institute, patients who have been identified with an anterior mediastinal mass all undergo physical examination and routine biochemical tests, an electrocardiogram (echocardiogram when indicated), chest X-ray, arterial blood gas analyses, and pulmonary function tests. A neurological protocol to exclude autoimmune diseases, particularly myasthenia gravis (MG) (21), is applied. After multi-slice computerized tomography (CT) scans (128 slices) are performed, the case is then discussed during multidisciplinary thoracic tumor board meetings together with a thoracic surgeon, pathologist, oncologist, anesthesiologist, radiotherapist, pneumologist, and chest radiologist. In case of indication to radical surgery, patients undergo cardiological, and pneumological evaluation of preoperative risk. Surgical indications are mainly based on patient clinical conditions and on the CT findings. Positron emission tomography (PET)–CT with fluorine-18 fluorodeoxyglucose (^18^F-FDG), magnetic resonance imaging (MRI), and octreotide scan are not part of the routine preoperative workup but are additional exams (22). When a complete resection is possible, preoperative biopsy is not indicated (23, 24). In case of invasion of adjoining structures such as the anonymous vein, pericardium, superior vena cava, phrenic nerves, and pleural cavities, a diagnostic biopsy is required; after the diagnosis by surgical biopsy or by fine needle biopsy aspiration (FNAB), the patient is usually referred to induction chemotherapy (25, 26) or to surgical treatment in combination with radiotherapy.

### Surgical Approach

Sternotomy and, in selected cases, thoracotomy represent the first surgical options because they allow an open extended resection of mediastinal masses and surrounding tissues, including mediastinal fat around the great vessels (27). However, in the last two decades, minimally invasive techniques took progressive place into clinical practice by a growing number of surgeons (28, 29). Minimally invasive techniques include video-assisted thoracoscopic surgery (VATS) (30) and robotic-assisted thoracoscopic surgery (RATS) (31). According to TYME, minimally invasive surgery is recommended for a tumor dimension smaller than 5 cm (17); however, also in case of invasion of neighboring organs (the pericardium, lungs, mediastinal pleura, or phrenic nerve), this procedure is not a contraindication in expert hands (23). The objective is quite similar for both RATS and VATS approaches: to perform standard extended thymectomy, including the thymus and the surrounding mediastinal fatty tissue, en bloc.

### Biobank: A Bridge Among Clinical and Scientific Resources

**Figure 1:**
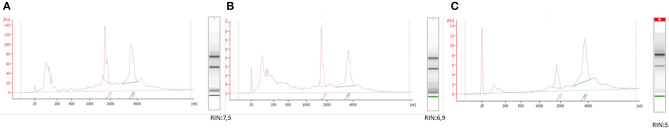
Methods in biobank: ID 528090BIOSPECIMEN QUALITY CONTROL. The image shows the quality of the different TET samples after RNA extraction using the different processing protocols **(A)** snap-frozen, **(B)** OCT, and **(C)** FFPE. The panel shows representative electropherograms of each sample type. The RIN values from each category were different between the groups (protocols A and B produced RIN values of ≥5, protocol C produced RIN values of ≤ 5—only moderately degraded RNA). All three methods guarantee the integrity of the RNA, rendering it suitable for most types of downstream applications.

The process of biobanking starts once a patient suspected of having mediastinal masses for thymic malignancy is identified and gives his/her institutional review board (IRB)-approved informed consent to preserve samples in our biobank. The consent is signed by both the patient and surgeon. A request for banking biological fluids is prepared prior to the surgical intervention by the surgeon through the creation of a computerized order entry to the Biological Fluids Biobank in the Clinical Pathology Laboratory. Blood samples (whole blood, serum, and plasma) are withdrawn by research nurses in the surgical ward (prior to operation and during follow-up to outpatients). The sterile tissue specimen is immediately collected from the operating room and taken to the Tissue Biobank in the Pathology Department upon removal. After checking and testing for biomaterial conformity and adequacy for diagnosis, sampling is performed by a “dedicated” pathologist (32). Each specimen is sampled depending on size and quality of the tissue; consecutive samples are prepared. The selected samples are immediately snap-frozen in liquid nitrogen or are frozen in optimal cutting temperature (OCT) and stored at −80°C. The procedure applies to both resected surgical specimens and biopsies (when sufficient material is available). Representative corresponding samples like morphological controls from either the tumor or the peritumoral thymus—when available—are fixed in formalin overnight (at 4°C) (minimum 24 h) and embedded in paraffin [formalin-fixed, paraffin-embedded (FFPE) material] (33) in a specific biobank archive. Tissue specimens are processed and stored in our tissue biobank by our “biobankers” according to the biobank standard operating procedures (SOPs) compliant with ISO9001/2015 certification (20). For sample collection and storage, clinical and biological data are recorded and managed by a dedicated software EasyTrack2D® according to the specific biobank SOPs. The quality of different fractions/samples (snap-frozen/OCT frozen/FFPE) is periodically evaluated for the preservation and yield of the cellular components by checking RNA/DNA extracted with RNA integrity number (RIN) (34). Figure 1 shows the RIN value of some of our sample RNAs. Recently, our biobank group has introduced the collection and isolation of tumor cells from fresh tumor specimens/neoplastic effusions (35). As for TET, we are setting up primary tumor cell cultures (preliminary data, not shown).

### Pathology—Diagnostic Workup and Digital Imaging

The recommendations of C.A. Moran and S. Suster (36) and of the International Collaboration on Cancer Reporting (ICCR) (37) in tumor sampling are followed (one tissue sample per centimeter of tumor or a minimum of 10 blocks for very large tumors). The peritumoral thymus is investigated by a “dedicated” pathologist who accurately and thoroughly examines the specimen and performs multiple sample embedding of peritumoral thymic fat tissue. In regard to pathological reporting, the 2015 WHO classification (8, 38) together with the ICCR recommendations (37) are followed. IHC plays a role in the diagnostic workup for diagnosis of thymomas with ambiguous histology and for the distinction between thymomas and thymic carcinomas (38). Pathological staging is performed by the pathologist on the basis of the tumor extent according to the eighth TNM (14, 39–41) published in its final and official version in 2017 (13). The Pathology Department, equipped with the Leica digital pathology platform Aperio AT2 (Aperio Leica Biosystems), performs most routine scans of representative slides of TET cases. Each H&E or significant IHC slide is scanned at a magnification of ×40. The scanning parameter settings are the default instrument settings. Digital images are analyzed by using the ImageScope® software. The image management system is the eSlide Manager® (12.3.3.5049) (Aperio Leica Biosystems).

### Research Pathway in TET

For molecular pathology, the methods applied in our tissue-based studies are only briefly mentioned here; the reader is referred to the original publications (18, 19, 42–44). We used sequencing and *egfr* fluorescence *in situ* hybridization (FISH) to genotype our series of thymomas: (I) for polymorphisms and somatic loss of heterozygosity of the non-coding *egfr* CA-SSR-1 microsatellite and (II) for *egfr* gene copy number changes. More recently, for our NGS study, we used the Ion AmpliSeq Cancer Hotspot Panel v2 targets 50, which is the most commonly used cancer panel adopted for solid tumors in order to identify mutations indicating sensitivity and resistance to targeted therapies. The panel is able to identify more than 2,800 COSMIC hot spots of 50 genes, as described in several studies (45, 46). For the microRNA study, microRNA expression profiling of FFPE tumor tissue and peritumoral thymus was performed by microarray analysis; mRNA expression profiling of fresh frozen TET and peritumoral thymus was performed by microarray analysis. The role of miR-145-5p in TETs was evaluated *in vitro*, modulating its expression in a thymic carcinoma (1889c) cell line. The epigenetic transcriptional regulation of miR-145-5p was examined by treating the cell line with the HDAC inhibitor valproic acid (VPA) (19).

## Results

Between 2001 and 2019, 196 cases of TET were recorded, excluding non-neoplastic thymic disease cases, in adult patients. The data reported in Table 1A exclude lymphoid neoplasias occurring or involving the thymus, such as Hodgkin lymphomas as well as non-Hodgkin lymphomas and the relatively common metastatic disease to the thymus/anterior mediastinum. Primary non-epithelial as well as non-lymphoid tumors were rarely diagnosed in the thymus (47). Table 1A briefly reports basic demographical data and subtype distribution of 188 TET cases seen at our institution. A slight increase in cases per year was recorded from 2016 (Table 1B). Most of the TET cases were surgically treated at our Institute. Cases involving second opinions were also included. Most of them derived from regions of Central or Southern Italy and were shared for second opinion diagnostic purposes from the Rare Cancer Center of the Regione Campania (CRTR). However, recently, cases referred to the NCI in Milan (INT), within the TYME network, were also shared with us and examined for a second opinion. Our Institute is a participating reference center both for diagnostic activity on TET in Italy within the TYME network (17) and for the pathological assessment of cases within a biological translational study (BIOTET) designed by the NCI in Milan (48).

**TABLE 1A:** Distribution by sex and histotypes of TET cases according to the 2015 WHO classification in the period 2001–2019—TET PATIENTS tot 196; TET, not further classifiable: 8 cases; Male: 100 (51%); Female:96 (49%).

**WHO histologic type (*n =* 188)**	
A	17 (9%)
AB	47 (25%)
B1	13 (7%)
B2	61 (33%)
B3	12 (6%)
Thymic carcinoma	38 (20%)

**TABLE 1B:** TET case number/year in the last years.

**Year**	**TET Total case no**.
2014	14
2015	11
2016	24
2017	26
2018	17
2019	18

Most cases, including those referred for a second diagnostic opinion and treated at IRE, are evaluated and discussed at the multidisciplinary thoracic tumor board (49). At our Institute, we apply consolidated surgical procedures, thymectomy being the cornerstone surgical approach used for treating patients with TET. According to international guidelines, the open approach is the first choice (23, 27); however, VATS and RATS (Table 2) also play a relevant role in our approach to thymic surgery. In our clinical practice, we routinely perform the RATS left approach for left-sided and central mediastinal lesions and reserve the right approach for right-sided tumors. The main advantages of this type of technique include the three-port access through 1-cm incisions, CO_2_ inflation in the mediastinum that radically increases operating space, accuracy of instrument movement under mechanical control, and 2D stereoscopic full-HD vision. Moreover, in the last few years, we have moved on from using the three-port VATS to the uniportal VATS. In comparison to RATS, the uniportal VATS approach, used only for small lesions with no invasion to adjacent structures (50, 51), even though slightly less accurate, has direct control over surgical instruments, returning to the tactile feedback of the surgeon's hand. Moreover, the uniportal access technique shows relevant post-operative pain reduction and better aesthetic results in comparison to the open approach.

**TABLE 2:** Distribution/year of thymectomies by RATS at IRE in the period 2016–2019.

	**2016**	**2017**	**2018**	**2019**
Robotic thymectomy	9	12	13	12

### Pathological TET Evaluation and Research Activities

For tumor diagnosis, classification, and digital imaging, in all cases, surgical specimens as well as bioptic material are classified according to the 2015 WHO classification, and the B2 subtype was the most represented histotype (33% of recorded cases) (Table 1A). Tumor tissue is routinely extensively sampled, and even though the amount of lymphocytes and/or thymocytes might vary in different areas of THY, the histological variation does not affect the main TET subtyping, performed according to the criteria set out in the 2015 WHO classification (8, 38). Moreover, extensive sampling allows the availability of FFPE material not only from the tumor itself but also from the peritumoral thymus, whenever remnant tissue is available. We provide blocks with “key-blocks” in order to evaluate the tumor and its surrounding tissue for accurate staging (37). Anterior mediastinal lymph nodes are also included in the sampling, because they are usually removed by the surgeons together with the fat tissue of the anterior mediastinum (52). In surgically treated THY cases at IRE, we found a metastasis in only one case, in a laterocervical lymph node (53), which developed 9 years from the original diagnosis. Recently, the use of the digital pathology is growing at an exponential rate, and we have been scanning most of the representative slides.

Tissue-based research activity in TET at IRE was first based on a tissue microarray (TMA)-based immunohistochemical study of vascular endothelial growth factor receptors (VEGFR family) in 200 cases from different Italian institutions. The TMA study provided evidence that tissue receptors of the VEGFR family are distributed among TET subtypes, reaching the maximum expression in TC (18). Subsequently, in a pilot study carried out on the *egfr* microsatellite CA-SSR-1 performed by the first genetic analyzer available in pathology, Thermo Fisher's 3130 genetic analyzer, we were able to show that CA-SSR-1 allelic imbalance with short allele relative prevalence significantly correlated with EGFR 3+ immunohistochemical scores, increased *egfr* gene copy numbers, and advanced stage with relapsing/metastatic behavior in thymomas (44). More recently, we have established further collaborations with other in-house research units (43) and national (19, 42, 54) and international institutes (55, 56). Thanks to frequent participation in meetings and interfacing with members of the scientific community at major conferences on thymic tumors, as well as holding structured workgroups supported by the scientific society ITMIG, our boundaries have changed and widened. The TCGA-THYM study participation is an example of a major cornerstone. This study, among other results, demonstrated the existence of four molecular subtypes in TET, which corresponded to the morphological subtypes in the WHO classification (57). In-house, we started an NGS study in order to map the genomic alterations of our TC series; preliminary data were presented at the most important conferences held on TET or at IASLC WCLC (58, 59).

**Figure 2:**
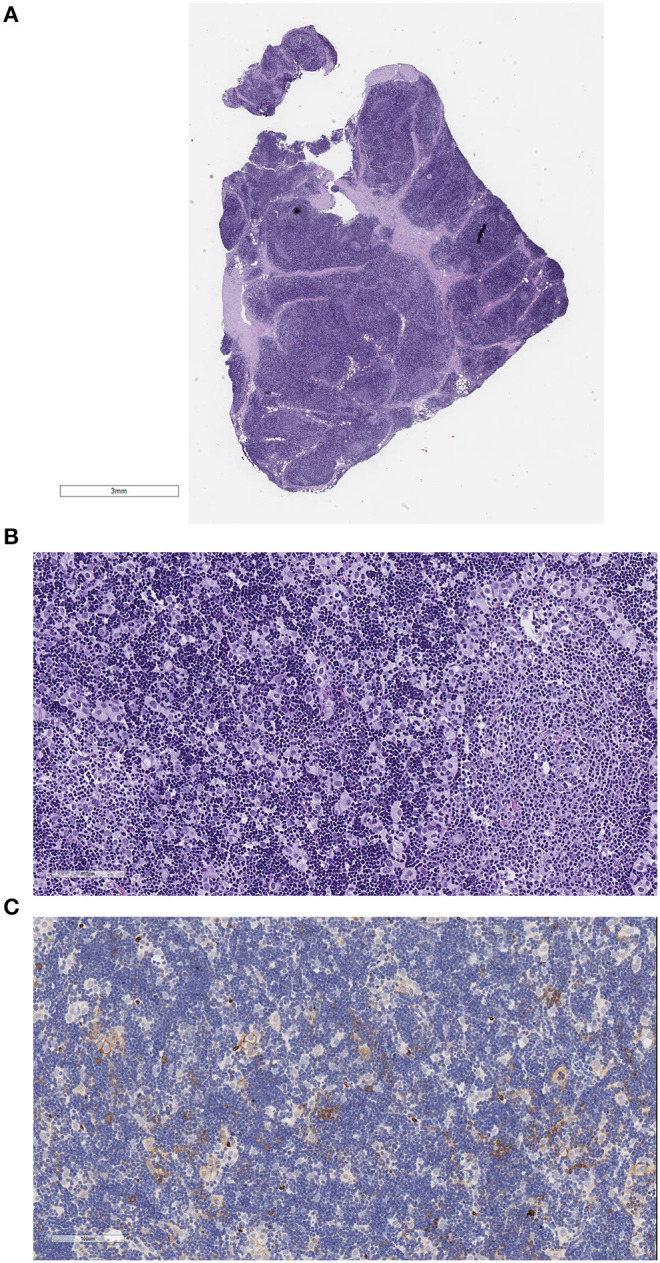
Example of a B2 thymoma fixed in formalin at 4°C and embedded in paraffin. The slides were scanned with the Aperio system 40×. Good morphological details are observed. **(A)** Hematoxylin–eosin (HE) stain, low magnification to show the whole section present on the scanned slide. **(B)** HE stain, 200×, showing the cortex-like tumor rich in epithelial cells (EC) and in thymocytes and a medullary island mostly containing lymphocytes. **(C)** Glut-1 stain of the B2 thymoma. Only few epithelial cells react.

**Figure 3:**
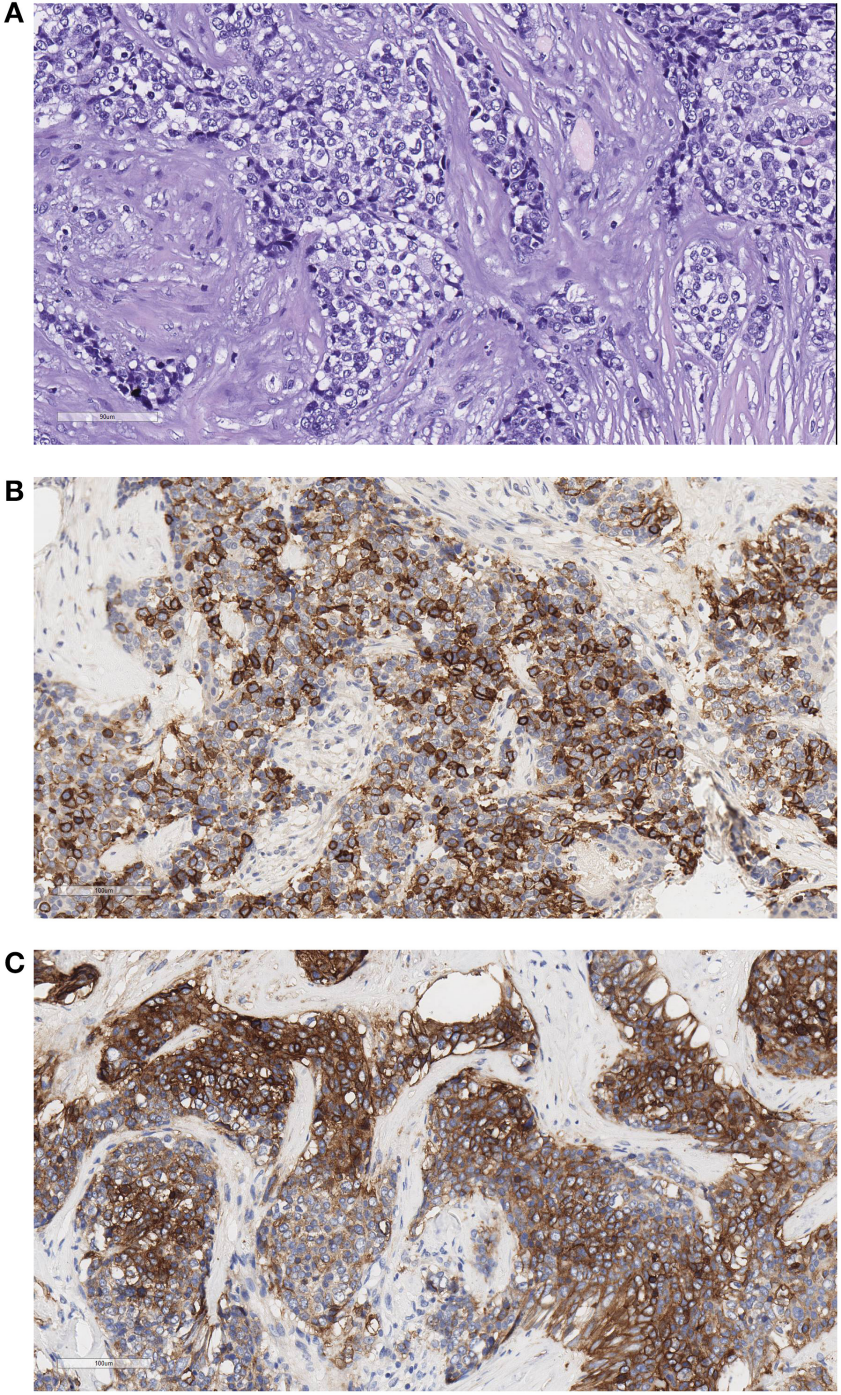
Example of a thymic carcinoma fixed in formalin at 4°C and embedded in paraffin. The slides were scanned with the Aperio system 40×. Good morphological details are observed. **(A)** Hematoxylin–eosin (HE) stain, 200×, showing the atypical epithelial cells forming ribbons infiltrating sclerotic tissue. **(B)** CD117 stain, 200×. Most cells are stained with this thymic carcinoma marker. **(C)** Glut-1 stains in thymic carcinoma ribbons and networks of epithelial cells.

For biobanking and TET frozen tissue-based research (60), the Thoracic Surgery Unit and the Pathology Department between September 2017 and May 2019 provided our biobank with tumor tissues from over 241 patients with thoracic tumors, including the most common lung carcinoma; TET; mesothelioma; and thoracic lymphoma (Table 3). At present (02/2020), we have 263 stored tumor samples from 31 patients affected with TET. The tumor samples preserved as morphological control and fixed in formalin at 4°C provided better morphological results than routine specimens (Figures 2, 3) (61). In the same period, at the Biological Fluid Biobank, we started to collect peripheral blood (PB) and serum/plasma from TET patients, thus preserving in the biobank complete samples (tumors and germline tissue) from 26 TET patients. Moreover, in the last few years, also before establishing our institutional biobank, we provided high-quality material from our “frozen collection of cases” to a gene expression profile carried out in our national scientific collaboration on microRNA. By analysis of a series of TET samples and peritumoral thymus, we identified a 69-gene signature of miR-145-5p putative target mRNAs. These mRNAs are differentially expressed between tumor and peritumoral thymus, and their expression is inversely correlated to that of miR-145-5p. Moreover, we evidenced that the epigenetic treatment of TC cell line 1889c with VPA, a histone acetylation inhibitor, resulted in the induction of miR-145-5p expression and downregulation of its target genes, showing antitumor effects in TET (cell cycle arrest and reduction of cell viability, colony-forming ability, and migration capability) (19).

**TABLE 3:** List of samples collected from 2017 to 2019 in our Biobank deriving from thoracic tumors.

			**Sample preservation mode**					
**Department**	**Pathology**	**Patients**	**Tumor tissue cryopreservation**	**Peritumoral tissue cryopreservation**	**Tumor tissue OCT**	**Peritumoral tissue OCT**	**FFPE**	**Total**
Thoracic surgery	Thymoma	22	169	49	6	4	20	248
	Lung tumors	152	801	711	65	39	130	1,746
	Mesothelioma	2	8	0	1	0	1	10
	Lymphoma	8	38	4	1	0	8	51
	Pleural effusion	35	0	0	0	0	0	0
	Peripheral blood (pleural effusion)	22	0	0	0	0	0	0
Total		241	1,016	764	73	43	20	2,055

*In addition to other thoracic tumors, specifically, 248 TET tissue samples from 22 patients were collected, of which 169 samples of snap frozen tumor tissue, 49 samples of adjacent normal snap frozen tissue. Moreover, we collected 6 samples of tumor tissues preserved in Optimal cutting temperature (OCT) and 4 samples of adjacent “peritumoral thymus” stored in OCT*.

## Discussion

The thymus is a primary lymphatic organ which sees the beginning of thymic involution at puberty (62), yet seeding, in an adult age, epithelial tumors deriving from highly specialized cells (63) of fundamental biological and pathological relevance. Our Institute has a long-standing interest in the diagnosis of thymic and mediastinal lesions (8, 38, 47) and management of TET patients (26, 42). Moreover, our Institute is well-known as an Italian expert center for the surgical and multimodality approach applied for the removal of mediastinal masses (25). At present, we play an active leading role in TYME, the main Italian network for thymic tumor management (17, 64), and we will be contributing to the ongoing ninth TNM staging project of thymic tumors and lung carcinoma, expected in 2024 (65, 66). In EURACAN, the G8 network, we contribute to ongoing activities in the clinical patient management system (CPMS), a web-based complex clinical software, and to the Digital Pathology Task force, and research projects are moving forward (16); currently, EURACAN in conjunction with the European Organization for Research on Cancer (EORTC) are moving ahead. EORTC, through SPECTA, an academic translational research infrastructure for biomaterial collection, aims to promote a comprehensive molecular profiling and virtual central pathology review also in the field of rare thoracic tumors.

### Translational Research Perspective

In our experienced clinical setting, over the past few years, we have applied multiple approaches toward TET tissue-based research studies. The TET biological system requires particular attention due to the occurrence of strictly intermingled epithelial and lymphoid cells in tumors. Therefore, IHC shows advantages because cells labeled with biomarkers are singularly identified. In our multicenter study on a series of 200 TET cases collected in the larger TET-TMA series built up, an extensive immunohistochemical angiogenesis-related investigation showed that VEGFR expression was associated with invasiveness and advanced stage (18). These data could provide biological support for the use of anti-angiogenetic drugs in TET treatment (67, 68). An Italian clinical trial exploring the role of angiogenetic receptors in TET is currently in progress (48).

Molecular and genomic studies, on the other hand, require attention in using TEC-enriched samples. In our pilot study focusing on the *egfr* relevance in the pathogenesis of TET, we provided statistically significant insight on the possible role that the length of the *egfr* microsatellite CA-SSR-1 and the *egfr* gene copy number could play in TET growth (44).

Subsequently, we established a successful collaboration with our Oncogenomic and Epigenetic Unit together with the Sapienza University of Rome, where we approached the epigenetic control of TET by microRNA-focused studies. First, we approached this field by using FFPE materials (43); then circulating microRNAs were investigated (54); subsequently, we contributed high-quality biobank-derived frozen material, allowing the gene expression profile of the mRNA putative target of miR-145-5p (19). We also started to perform the functional characterization of the 1889c cell line (60) by investigating the epigenetic regulation of miR-145-5p, as well as the modulation of its functional target mRNAs in our system. Of note, we are now engaged in the characterization of the contribution of the long non coding RNA (lncRNA) function in TET. Very few reports so far investigated lncRNA in TET (69). We are focusing our attention on the sponge activity of lncRNAs, which are able to inhibit the microRNA function generating molecular networks relevant for tumor establishment and progression. Our preliminary data (not shown) highlight the relevance of the epigenetic deregulation of ncRNA in TET for the identification of novel molecular targets of therapy.

The quality of our biobank material was also confirmed by the inclusion of our samples among the cases included in the TCGA-THYM study (57). Recently, we have focused on implementing our biobanking activities. These were supported by a strategy based on a positive feedback cycle between the thoracic surgeon and the “dedicated” pathologist, by the development of an efficient and certified biobanking system, and by the implementation of laboratory cell culture facilities. In fact, our purpose now is to set up a procedure for the isolation of stem cells from fresh TET specimens, based on our previous experience in different tumor systems (70). Preliminary data on primary cultures of TET appear to be promising (data not shown). In the field of imaging analysis, digital pathology is a rapidly evolving and increasingly utilized tool in histology. It enables high throughput and precise analysis of a large number of samples and facilitates easier interactive consensus in remote diagnostic discussions, as we achieved in the TCGA-THYM study (57). TCGA deriving image archives—otherwise underutilized—recently provided insight into the tumor-immune microenvironment in 13 TCGA tumor types (71). All the studies reported a major role played by the “dedicated” pathologist. The role of pathologist evolved from giving microscopic description to adhering to internationally validated classification criteria (38) and to adopting structured pathology reports (37) in order to provide standardized and relevant information for prognostic stratification of patients. The pathologist also plays a major role in identifying new biomarkers by IHC; digitized slides provide quantitative as well as qualitative observations. Moreover, the morphological evaluation of tumor samples for molecular analyses prevents inadequate sampling and inappropriate molecular analyses on necrotic or fibrotic tissue. Bridging the gap between molecular data and the knowledge of the biological/tumoral systems, the pathologists contribute to integrating morphology with molecular findings. Based on our examples above, it is evident that solid commitment from the Pathology Department is critical for translational research and in all aspects of clinical care, especially in rare tumor types.

The challenging points of our well-established study on TET and of tissue-based translational studies range from the limited availability of cases and funding to the difficulties in clinical data collection. Moreover, given the specific biology of TET, outcome indicators are difficult to collect due to the long natural history of thymomas and to the possibilities of patients migrating or returning to their place of origin, being lost to follow-up. Clinical trials for TET (48) are difficult to promote and to find collaborative support from pharmaceutical companies, as these tumors are orphan diseases (10). Currently, at our institute, new TET cases are discussed at our multidisciplinary thoracic tumor Board meetings (49, 72) as they are an important tool in achieving the best approach to patient management. Our Institute routinely performs second opinion pathological review for the majority of patients who seek oncologic consultations. A second look in specialized centers for rare tumors can result in major prognostic and therapeutic modifications (73). Despite the limited funding for our translational research projects on TET, we have received free support from our research collaborating units who have contributed in providing reagents, human resources, and the use of their platforms. This type of eager collaborative support happens when there is a deep-seated belief in a type of rare tumor that is deserving of attention and interest. At the same time, health networks such as EURACAN provided improvement in patient assistance (74) and are expected to promote translational research in rare tumor.

Therefore, although our clinical responsibilities have been greatly burdened over the last few years, we, as a team, have set the grounds for significantly contributing scientifically to TET research. We hope to implement our translational research activity by improving our networking with other research centers in both Italy/Europe and abroad. In the future, translational research will offer precision medicine data and targeted therapies to the clinical management of TET patients.

## Data Availability Statement

The datasets generated for this study are available on request to the corresponding author.

## Ethics Statement

This study was carried out in accordance with the recommendations of our Ethical Committee. The protocol was reviewed and approved by the Comitato Etico Centrale IRCCS Lazio -Found. Bietti. All subjects gave written informed consent in accordance with the Declaration of Helsinki.

## Author Contributions

MM designed the manuscript and drafted it. EM, EG, SM, FG, VL, GA, and FFaz participated in the designing and drafting up of the manuscript. GB and NG critically revised it. EP, FFac, LC, and GC coordinated the manuscript. Administrative support was given by EP. All the authors contributed to the work during the years by their clinical or experimental activity. All authors contributed to the article and approved the submitted version.

## Conflict of Interest

The authors declare that the research was conducted in the absence of any commercial or financial relationships that could be construed as a potential conflict of interest.
